# Medication prescribing and pregnancy-related risk factors for women with type 2 diabetes of reproductive age within primary care: a cross-sectional investigation for the PREPARED study

**DOI:** 10.1136/bmjdrc-2024-004312

**Published:** 2025-03-05

**Authors:** Alexandra M Famiglietti, Judith Parsons, Kia-Chong Chua, Anna Hodgkinson, Olubunmi Abiola, Anna Brackenridge, Anita Banerjee, Mark Chamley, Lily Hopkins, Katharine F Hunt, Helen Murphy, Helen Rogers, Gavin Steele, Kirsty Winkley, Angus Forbes, Rita Forde

**Affiliations:** 1 Florence Nightingale Faculty of Nursing, Midwifery & Palliative Care, King's College London, London, UK; 2 Institute of Psychiatry, Psychology and Neuroscience, King's College London, London, UK; 3 Department of Diabetes and Endocrinology, Guy's and St Thomas' Hospitals NHS Trust, London, London, UK; 4 NHS South East London Integrated Care Board, London, UK; 5 PPI Member, c/o Faculty of Nursing, Midwifery and Palliative Care, King's College London, London, UK; 6 Women’s Health Services, Guy's and St Thomas' Hospitals NHS Trust, London, London, UK; 7 North Wood Group Practice, London, UK; 8 London School of Hygiene & Tropical Medicine, London, UK; 9 Diabetes Department, King's College Hospital NHS Foundation Trust, London, UK; 10 University of East Anglia Norwich Medical School, Norwich, UK; 11 Nexus Health Group, London, UK; 12 School of Nursing and Midwifery, University College Cork, Cork, Ireland

**Keywords:** Type 2 Diabetes, Contraception, Pregnancy, Primary Health Care

## Abstract

**Introduction:**

Women with type 2 diabetes are at risk of commencing pregnancy while using medications that are either not recommended for pregnancy or with known teratogenicity, which may contribute to adverse pregnancy outcomes. In this study, we aimed to characterize pregnancy-related risk factors and medication exposures among women with type 2 diabetes.

**Research design and methods:**

Individual health characteristics, sociodemographic information, and prescription data were extracted from the primary care records of women aged 18–45 years with type 2 diabetes in participating general practices in the UK. Prescribed medications were categorized according to suitability for pregnancy: recommended, not recommended, or not recommended but used if clinically indicated. Logistic regression was used to estimate associations between individual characteristics and medications not recommended for pregnancy.

**Results:**

Data on 725 women were extracted. Prescribed medications suggested the presence of numerous comorbidities, with diabetes medications (65%, n=471) and statins (20%, n=145) most frequently prescribed. 37% (n=268) of women took ≥3 medications, and a third (n=269) took medications not recommended for pregnancy. Among those not prescribed contraception (89%, n=646), no one met all clinically recommended pre-pregnancy criteria. In multivariable logistic regression analysis, polypharmacy (OR 3.49 95% CI 2.88 to 4.30) and age (OR 1.04 95% CI 1.00 to 1.09) were associated with use of medications not recommended for pregnancy.

**Conclusions:**

Women with type 2 diabetes have suboptimal contraceptive provision despite multiple exposures to medications not recommended for pregnancy. Regular assessment of contraceptive use, reproductive intentions, and medication review is urgently needed in primary care settings to minimize pregnancy-related risks.

**WHAT IS ALREADY KNOWN ON THIS TOPIC**

- In the UK, only 1 in 10 women with type 2 diabetes is adequately prepared for pregnancy, a figure significantly lower than that observed among women with type 1 diabetes.
- A substantial number of women with type 2 diabetes are likely to commence pregnancy while using medications not recommended for pregnancy or with known teratogenicity, which may contribute to adverse pregnancy outcomes. Documentation of the medication profile and contraception use of this population is limited.

**WHAT THIS STUDY ADDS**

- Only 1 in 10 women was prescribed contraception by their general practitioner, suggesting that the potential of unplanned pregnancies in this population is high.
- Over one third of women use a medication not recommended for pregnancy, and concordance with clinically recommended pre-pregnancy criteria is low.
- The odds of the prescription of medications not recommended for pregnancy are higher among older women and among women using multiple medications.

**HOW THIS STUDY MIGHT AFFECT RESEARCH, PRACTICE OR POLICY**

- In primary care settings, regular assessment of contraceptive use, evaluation of reproductive intentions, and corresponding medication review is urgently needed to minimize the pregnancy-related risks of women of reproductive age with type 2 diabetes.

## Introduction

 Appropriate pregnancy preparation for women with type 2 diabetes reduces the risk of adverse pregnancy outcomes, including birth defects and perinatal death.^1^,^3^ Clinical guidance recommends diabetes-specific care and medication review prior to pregnancy to promote the early identification of modifiable risk factors.^4^ Pre-pregnancy guidelines for women with type 2 diabetes developed by the National Institute for Care and Health Excellence (NICE) specifically recommend prior to conception the cessation of statins, ACE inhibitors, and angiotensin-II receptor blockers; the provision of high dose (5 mg) folic acid; and the fulfillment of specified targets for glycated hemoglobin (HbA1c, <48 mmol/mol or 6.5%) and body mass index (BMI, <27 kg/m^2^).^4^ Nevertheless, compared with women with type 1 diabetes, women with type 2 diabetes are more likely to commence pregnancy while taking medications not recommended for pregnancy or with known teratogenicity.^2^ This is partly a consequence of the types of medications commonly used to manage type 2 diabetes, in addition to the fact that women in the UK with type 2 diabetes are managed in primary care settings, whereas women with type 1 diabetes are managed in specialist diabetes services that provide pre-pregnancy support. This trend is increasingly concerning, as type 2 diabetes currently represents 54% of pregnancies in women with pre-existing diabetes in the UK, and this proportion is steadily rising.^2^

In a systematic review investigating factors associated with the provision and uptake of pre-pregnancy care in women with type 2 diabetes,^5^ we identified a lack of knowledge and awareness of adequate preparation for pregnancy with diabetes among healthcare professionals and women living with this long-term condition. This deficit is exacerbated by the management of women with type 2 diabetes in UK primary care settings, where the delivery of reproductive health, including pre-pregnancy care, is currently not incorporated in routine diabetes management.^6^ Consequently, women’s opportunities to access appropriate and timely reproductive health support are ad hoc, increasing the risk of unplanned pregnancies. This trend is reflected in national diabetes pregnancy audit data, which reveal that only 1 in 10 women with type 2 diabetes is adequately prepared for pregnancy.^2^ The national audit further indicates that 12% of women use potentially harmful medications (ACE inhibitors, statins, and other adverse diabetes medications) before pregnancy, increasing the risk of adverse fetal exposure during organogenesis, a critical phase of pregnancy.^7 8^ Delayed referrals to specialist diabetes maternity clinics among women with type 2 diabetes as compared with women with type 1 diabetes further contributes to the risk of congenital malformations caused by deleterious medication exposure.^7^ To ameliorate these risks, medication review should be undertaken prior to conception.

In this study, we present an observational analysis of data collected from all women of reproductive age (18–45 years), irrespective of pregnancy intention or potential, with type 2 diabetes from participating general practices in South London using baseline data from our pre-pregnancy intervention study: *An integrated primary care-based programme of PRE-Pregnancy cARE to improve pregnancy outcomes in women with type 2 diabetes* (the PREPARED study).^9 10^ The aim of this analysis was to characterize the pregnancy-related risk factors and medication exposures among the specified cohort. Specifically, the objectives of this investigation were to:

- Identify the proportion of women prescribed hormonal contraception or a documented intrauterine device (IUD) by their general practitioner (GP).
- Describe the medication profile of the cohort, with particular focus on hypoglycemic agents, drug types, polypharmacy, and drugs not currently recommended for pregnancy.
- Determine the proportion of the cohort not using contraception prescribed by their GP who meet specified NICE pre-pregnancy criteria, including medication use.
- Model associations between sociodemographic and clinical factors, exposure to medications not recommended in pregnancy, and NICE pre-pregnancy criteria.

## Methods

### Data collection and study population

Between September and October 2022, data were extracted using a bespoke electronic audit tool from the EMIS electronic record keeping system of 29 GP practices in two diverse South London boroughs (Lambeth and Southwark). Data extraction captured sociodemographic and medical data from the records of individuals registered in participating GPs that met the following criteria: woman, aged 18–45 years, recorded type 2 diabetes diagnosis, and no recorded gynecological surgeries such as hysterectomy or oophorectomy. Information on both age and ethnic origin were collected, and ethnic origin was stratified according to the Office for National Statistics Census 2021 ethnic group classification 6a: (1) Asian, Asian British, or Asian Welsh, (2) Black ethnicity, Black British, Black Welsh, Caribbean, or African, (3) mixed or multiple ethnic groups, (4) white ethnicity, and (5) other ethnic group, declined to respond, or missing.^11^ Medical data included the most recently recorded measurements of glycemia (HbA1c), renal function, blood pressure, cholesterol, and BMI for each eligible individual. The details of all medications prescribed in the previous 12 months were extracted, with differentiations made between repeat prescriptions (used for ≥6 months) and single prescriptions for one-off courses of treatment. Staff in each of the participating GP practices anonymized all data before sharing it with the research team.

### Medication classification

Prescribed medications were sorted by drug class following British National Formulary (BNF) guidance; classifications were then reviewed by a team of two clinicians (RF and AF) and a specialist pharmacist (AH). Subsequent analyses considered only repeat prescriptions, excluding single courses of medications. Information on duration of prescription or medication adherence was not available. Medication groups considered unlikely to affect pre-pregnancy care (eg, topical ointments and medical materials) were also excluded. Polypharmacy was assessed by calculating the total number of medication classes prescribed to each individual, excluding folic acid and contraception prescriptions.

BNF recommendations were also used to determine if medications were suitable for use during pregnancy. Each medication was assigned to one of the following three groups based on its BNF drug profile:

1. Not recommended for pregnancy. These medications either have manufacturer-specific guidance to avoid use during pregnancy or existing evidence of potential harm during any stage of pregnancy. Drugs such as renin–angiotensin system (RAS) acting agents, statins, dipeptidyl peptidase-4 (DPP-4) inhibitors, and glucagon-like peptide-1 (GLP-1) inhibitors were included in this group.
2. Not recommended but used in pregnancy if clinically indicated. Definitive guidance is lacking and use of the medication during pregnancy is often advised to be evaluated on a case-by-case basis by the prescribing clinician.
3. Recommended for pregnancy. No evidence indicates that the medication could cause harm in pregnancy, and therefore there are no existing restrictions on its use in pregnancy.

The final list consisted of 178 repeatedly prescribed medications, of which 66 were designated as not recommended for pregnancy (online supplemental table S1).

### Pre-pregnancy criteria

To establish how well prepared for pregnancy women with type 2 diabetes were according to NICE guidance, we calculated the proportion of women not prescribed contraception by their GP against the following criteria: (1) BMI<27 kg/m^2^, (2) prescribed 5 mg of folic acid, (3) HbA1c<48 mmol/mol (6.5%), (4) not prescribed RAS-acting agents, and (5) not prescribed statins.^4^ While the pregnancy intentions of the women included in this audit were unknown, as a group with reproductive potential, the data were considered against the NICE criteria for optimal pre-pregnancy care for women with type 2 diabetes.

### Statistical analysis

The extracted data were reviewed and checked for accuracy. Descriptive statistics for sociodemographic and clinical characteristics, as well as prescriptions, were used to characterize the sample and to provide frequencies for the pre-pregnancy criteria and medication exposures. These data were tabulated detailing profiles for those who were and were not prescribed contraception by their GP. The following two multivariable logistic regression analyses were undertaken using R statistical software (V.4.2.2, R Foundation for Statistical Computing):

- Model 1 investigated the association between clinical factors (most recent systolic and diastolic blood pressure, BMI, and HbA1c measurements) and the prescription of contraception in the past 12 months by their GP, adjusting for sociodemographic factors (age and ethnic origin).
- Model 2 explored the association between clinical factors (most recent systolic and diastolic blood pressure, BMI, and HbA1c measurements) and prescription of medications not recommended during pregnancy, adjusting for sociodemographic factors (age and ethnic origin). Polypharmacy was also included in this model as a count variable. Women prescribed contraception by their GP were excluded, as they were considered at low risk of pregnancy.

In both models, clinical characteristic variables were dichotomized based on clinical thresholds for BMI (25 kg/m^2^)^12^ and HbA1c (48 mmol/mol or 6.5%)^4^; estimated glomerular filtration rate (eGFR) and cholesterol were excluded from the models, as there were inadequate numbers of women in the higher risk categories (eGFR<6 mL/min/1.73 m^2^ and total cholesterol>5 mmol/L). Additional clinical variables (including urine albumin to creatinine ratio) with widespread missing data were not considered for model inclusion.

## Results

### Participant sociodemographics

In total, we accrued data on 725 women of reproductive age (18–45 years) with type 2 diabetes, with each practice (n=29) contributing between 9 and 48 eligible participants. The median age of the cohort was 38 years (IQR 6.4), half of the women were aged 40–45 years (50%, n=362), and nearly half (45%, n=326) were of Black ethnicity. The majority of the women were overweight (18%, n=131) or obese (72%, n=522). Of those who were obese, 23% (n=167) had a BMI≥40 kg/m^2^. With regard to glycemia, 29% (n=209) of the cohort had a HbA1c below 48 mmol/mol (6.5%), while the remaining women exceeded the current NICE pre-pregnancy target, with 17% (n=122) having a HbA1c≥86 mmol/mol (10%). The full sociodemographic and clinical characteristics of the cohort are detailed in table 1.

**Table 1:** Cohort sociodemographic and health characteristics by contraception use

	Contraception(n=79)	No contraception(n=646)	Total(n=725)
Sociodemographic characteristics			
Age (years, median, IQR)	38.2 (5.8)	37.7 (6.5)	37.7 (6.4)
Age (categorized)			
18–25 years	1 (1.1)	28 (4.3)	29 (4.0)
25–29 years	7 (8.9)	58 (9.0)	65 (9.0)
30–34 years	8 (10.1)	104 (16.1)	112 (15.4)
35–39 years	20 (25.3)	136 (21.1)	156 (21.5)
40–45 years	43 (54.4)	319 (49.4)	362 (49.9)
NA	0	1 (0.2)	1 (0.1)
Ethnic origin			
Asian/Asian British	10 (12.7)	121 (18.7)	131 (18.1)
Black ethnicity/African/Caribbean/Black British	26 (32.9)	300 (46.4)	326 (45.0)
Mixed/multiple ethnic groups	11 (13.9)	59 (9.1)	70 (9.7)
White ethnicity/White British	23 (29.1)	110 (17.0)	133 (18.3)
Does not apply/other	9 (11.3)	56 (8.7)	65 (9.0)
Health characteristics			
ACR (g/mol, median, IQR)	1.1 (1.4)	1.1 (2.0)	1.1 (1.9)
BMI (median, IQR)	35.0 (11.0)	33.0 (10.0)	34.0 (10.0)
BMI (categorized)			
< 25 kg/m^2^	4 (5.1)	57 (8.8)	61 (8.4)
25–29 kg/m^2^	18 (22.8)	113 (17.5)	131 (18.1)
30–34 kg/m^2^	17 (21.5)	188 (29.1)	205 (28.3)
35–39 kg/m^2^	19 (24.1)	131 (20.3)	150 (20.7)
≥ 40 kg/m^2^	21 (26.6)	146 (22.6)	167 (23.0)
Blood pressure (mm Hg, median, IQR)			
Systolic arterial pressure	124.0 (21.0)	123.5 (20.0)	124.0 (20.0)
Diastolic arterial pressure	79.0 (12.5)	80.0 (12.0)	80.0 (12.0)
Cholesterol (mmol/L, median, IQR)			
Total	4.6 (1.5)	4.7 (1.3)	4.7 (1.4)
LDL	2.8 (1.3)	2.7 (1.2)	2.7 (1.2)
HDL	1.2 (0.4)	1.3 (0.4)	1.2 (0.4)
eGFR (mL/min, median, IQR)	90.0 (28.0)	90.0 (21.0)	90.0 (22.0)
eGFR (categorized)			
< 60 mL/min	3 (3.8)	20 (3.1)	23 (3.2)
60–89 mL/min	29 (36.7)	172 (26.6)	201 (27.7)
≥ 90 mL/min	47 (59.5)	438 (67.8)	485 (66.9)
HbA1c (median, IQR)			
mmol/mol	52.0 (25.0)	54.0 (28.0)	54.0 (27.5)
%	6.9 (4.4)	7.1 (4.7)	7.1 (4.7)
HbA1c (categorized)			
>48 mmol/mol (6.5%)	29 (36.7)	180 (27.9)	209 (28.8)
48–57 mmol/mol (6.5–7.4%)	17 (21.5)	199 (30.8)	216 (29.8)
58–69 mmol/mol (7.5–8.5%)	13 (16.5)	76 (11.8)	89 (12.3)
70–85 mmol/mol (8.6–9.9%)	9 (11.4)	70 (10.8)	79 (10.9)
≥86 mmol/mol (10.0%)	11 (13.9)	111 (17.2)	122 (16.8)

Values are presented as n (%) or median (IQR), with % indicating the proportion of women within the specified contraception category that exhibit the stated characteristic.

ACRurine albumin to creatinine ratioBMI, body mass index; eGFR, estimated glomerular filtration rate; HbA1c, glycated hemoglobinHDL, high-density lipoprotein; LDL, low-density lipoprotein

### GP-prescribed contraception

In the cohort, only 11% (n=79) of women had been prescribed contraception (oral, injectable, patch, IUD, etc) in the past 12 months by their GP. Model 1 estimated that the odds of women of Asian (OR 0.392, 95% CI 0.170 to 0.853, p=0.022) and Black ethnicities (OR 0.367, 95% CI 0.198 to 0.683, p=0.001) being prescribed contraception by their GP were significantly lower compared with women of white ethnicity (table 2). No other sociodemographic or clinical characteristics had a significant interaction in the model.

**Table 2:** Model 1: Association between contraception and cohort characteristics

DV: contraception (Y/N)	OR (95% CI)	P value
Age (years)	1.015 (0.977 to 1.057)	0.458
Blood pressure (mm Hg)		
Systolic≥140	1.150 (0.519 to 2.435)	0.721
Diastolic≥90	0.722 (0.358 to 1.426)	0.976
BMI≥25 (kg/m^2^)	0.989 (0.456 to 2.033)	0.160
Ethnic origin		
Asian/Asian British	0.392 (0.170 to 0.853)	0.022*
Black ethnicity/African/Caribbean/Black British	0.367 (0.198 to 0.683)	0.001*
Mixed/multiple ethnic groups/other	0.777 (0.398 to 1.505)	0.454
White ethnicity/white British (ref)		
HbA1c≥48 mmol/mol	0.661 (0.402 to 1.102)	0.106

Logistic regression model describing the association between the prescription of contraception by GPgeneral practitioner and stated sociodemographic and health cohort characteristics.

* p value<0.05.

BMIbody mass indexDVdependent variableHbA1cglycated hemoglobin

### Medications

Women were prescribed a wide range of medications that could suggest a diverse burden of comorbidities (table 3). Diabetes medications were the most frequently prescribed (65.0%, n=471), followed by antihypertensives (27.2%, n=197) and statins (20.0%, n=145). Prescribed diabetes medications included the following: metformin, 57.5% (n=417); insulin, 14.9% (n=108); secretagogues, 11.3% (n=82); DPP-4 inhibitors, 8.3% (n=60); sodium–glucose cotransporter-2 inhibitors, 7.9% (n=57); and GLP-1 inhibitors, 5.0% (n=36). Online supplemental table S2 details the profile of diabetes medications across HbA1c groups. Psychotropic medications, which mainly consisted of antidepressants, were prescribed to 13.9% (n=101) of women. Polypharmacy was observed throughout the cohort, with over a third being prescribed ≥3 medication groups and 12.7% (n=92) being prescribed ≥5 medications in the 12 months prior to data extraction. Notably, 37.1% (n=269) of women had been prescribed one or more medication not recommended for use during pregnancy, which reduced to 31.0% (n=225) when diabetes medications were excluded.

**Table 3:** Cohort medication profile by contraception use

	Contraception(n=79)	No contraception(n=646)	Total(n=725)
Medications			
Amenorrhea	0	10 (1.5)	10 (1.4)
Antibiotics	0	9 (1.4)	9 (1.2)
Anticoagulants	2 (2.5)	6 (0.9)	8 (1.1)
Anticonvulsants	0	7 (1.1)	7 (1.0)
Antihypertensives	27 (34.2)	170 (26.3)	197 (27.2)
Alpha-adrenergic agonists	0	1 (0.2)	1 (0.1)
Alpha-adrenergic blockers	0	5 (0.8)	5 (0.7)
Beta blockers	2 (2.5)	29 (4.5)	31 (4.3)
Calcium channel blockers	16 (20.3)	86 (13.3)	102 (14.1)
Diuretics	5 (6.3)	23 (3.6)	28 (3.8)
RAS-acting agents	19 (24.1)	109 (16.9)	128 (17.7)
Aspirin	2 (2.5)	16 (2.5)	18 (2.5)
Diabetes medications	54 (68.4)	417 (64.6)	471 (65.0)
DPP-4 inhibitors	9 (11.4)	51 (7.9)	60 (8.3)
GLP-1 inhibitors	7 (8.9)	29 (4.5)	36 (5.0)
Insulin	13 (16.5)	95 (14.7)	108 (14.9)
Metformin	49 (62.0)	368 (57.0)	417 (57.5)
Secretagogues	8 (10.1)	74 (11.5)	82 (11.3)
SGLT2 inhibitors	10 (12.7)	47 (7.3)	57 (7.9)
Gastrointestinal medications	14 (17.7)	95 (14.7)	109 (15.0)
Hormone replacement therapy	1 (1.3)	6 (0.9)	7 (1.0)
Pain management medication	3 (3.8)	35 (5.4)	38 (5.2)
Psychotropic medications	13 (16.5)	88 (13.6)	101 (13.9)
Antidepressants	13 (16.5)	75 (11.6)	88 (12.1)
Antipsychotics	0	17 (2.6)	17 (2.3)
Mood stabilizers	0	2 (0.3)	2 (0.3)
Stimulants	0	1 (0.2)	1 (0.1)
Statins	22 (27.8)	123 (19.0)	145 (20.0)
Thyroid medication	8 (10.1)	34 (5.3)	42 (5.8)
Polypharmacy			
No agents	18 (22.8)	160 (24.8)	178 (24.6)
1–2 agents	23 (29.1)	256 (39.6)	279 (38.5)
3–4 agents	21 (26.6)	155 (24.0)	176 (24.3)
≥5 agents	17 (21.5)	75 (11.60)	92 (12.7)
Use during pregnancy			
Not recommended Not recommended (excluding diabetes medications)	36 (45.6)28 (35.4)	233 (36.1)197 (21.2)	269 (37.1)225 (31.0)
Only if clinically indicated	37 (46.8)	258 (39.9)	295 (40.7)
Recommended	56 (70.9)	405 (62.7)	461 (63.6)

Values are presented as n (%), with % indicating the proportion of women within the specified contraception category that have been prescribed a medication in the stated class for six6 or more months. See Table S1online supplemental table S1 for the full medication list.

DPP-4, dipeptidyl peptidase-4; GLP-1, glucagon like peptide-1; RASrenin–angiotensin systemSGLT2, sodium–glucose cotransporter-2

The model 2 regression demonstrated that the odds of the prescription of medications not recommended for pregnancy were slightly higher among older women (OR 1.044, 95% CI 1.004 to 1.088, p=0.034) and among women using more medications (OR 3.49, 95% CI 2.88 to 4.30, p<0.001), when adjusted for specified sociodemographic and clinical variables (table 4). In contrast, the odds of the prescription of medications not recommended for pregnancy were lower among women with a HbA1c above 48 mmol/mol (6.5%, OR 0.447, 95% CI 0.264 to 0.750, p=0.002), the threshold recommended for optimal pre-pregnancy care. No association was evident with ethnic origin, blood pressure, or BMI. In a subanalysis excluding diabetes medications not recommended in pregnancy, the results were minimally impacted; age, HbA1c, and polypharmacy remained the only significant variables.

**Table 4:** Model 2: Association between the prescription of medications not recommended for pregnancy and cohort characteristics in women not prescribed contraception by their general practitioner

DV: Prescription of medication not recommended for pregnancy (Y/N)	OR (95% CI)	P value
Age (years)	1.044 (1.004 to 1.088)	0.034*
Blood pressure (mm Hg)		
Systolic≥140	0.986 (0.453 to 2.118)	0.971
Diastolic≥90	0.722 (0.358 to 1.426)	0.354
BMI≥25 (kg/m^2^)	1.278 (0.545 to 3.104)	0.579
Ethnic origin		
Asian/Asian British	1.024 (0.455 to 2.316)	0.954
Black ethnicity/African/Caribbean/Black British	1.183 (0.602 to 2.371)	0.631
Mixed/multiple ethnic groups/other	1.512 (0.695 to 3.330)	0.230
White ethnicity/white British (ref)		
HbA1c≥48 mmol/mol	0.447 (0.264 to 0.750)	0.002*
Polypharmacy	3.486 (2.881 to 4.304)	<0.001*

Logistic regression model describing the association prescription of medications not recommended for pregnancy and stated sociodemographic and health cohort characteristics.

* p value<0.05.

BMI, body mass index; DV, dependent variable; HbA1c, glycated hemoglobin

### Concordance with NICE guidelines

Concordance with NICE pregnancy guidelines varied by criterion (table 5). Over 80% of the cohort did not use RAS-acting agents (n=537) and/or statins (n=523), while only 5.1% (n=33) of the population had been prescribed high dose folic acid. No women met all five specified criteria, and 4.2% (n=27) of the cohort met none of the criteria.

**Table 5:** NICE guideline concordance among women not prescribed contraception by their general practitioner

NICE criteria	No contraception (n=646)
BMI<27 kg/m^2^	180 (27.9)
Folic acid (5 mg) used	33 (5.1)
HbA1c<48 mmol/mol	180 (27.9)
RAS-acting agents not used	537 (83.1)
Statins not used	523 (81.0)
No criteria met	27 (4.2)
One criterion met	102 (15.8)
Two criteria met	296 (45.8)
Three criteria met	185 (28.6)
Four criteria met	36 (5.6)
All criteria met	0

Values are presented as n (%), with % indicating the proportion of women that fulfill the stated NICE criterion.

BMI, body mass index; HbA1c, glycated hemoglobin; NICENational Institute for Health and Care ExcellenceRAS, renin–angiotensin system

## Discussion

This cross-sectional study indicates that women aged 18–45 years diagnosed with type 2 diabetes in South London are exposed to several factors that increase their pregnancy risk profile. With only 1 in 10 women in this cohort prescribed contraception by their GP, the risk of an unplanned pregnancy was high, even allowing for the possible use of contraception accessed from other sources (reproductive health clinics or pharmacies). Although individual pregnancy potential and intention within this cohort was unknown, given the high incidence of unplanned pregnancy and persistently low receipt of pre-pregnancy care within this population, the risk of adverse pregnancy outcomes remains a significant concern.^13 14^

Analysis of participant medication profiles revealed that over one third of women use a medication not recommended for pregnancy. While the health benefits gained by the use of such medications may outweigh possible risks for some women in this population, the ubiquity of these prescriptions highlights the need for suitable strategies to minimize adverse pregnancy outcomes. Related strategies should facilitate increased type 2 diabetes pregnancy awareness, promote appropriate contraception, and streamline referrals to pre-pregnancy care when pregnancy is considered. It is important, therefore, that primary care practitioners regularly elicit and review women’s pregnancy intentions to either provide adequate contraception or, if pregnancy is sought, ensure early referral to pre-pregnancy care services for medication management based on individual health needs. The level of exposure to potentially teratogenic medications observed in this cohort of women, even with diabetes medications excluded, was markedly high (31%). While comparative data are limited, a large primary care cohort study (n=1000) recently reported that among women of reproductive age, 95% of whom did not have diabetes, only 17% had been prescribed medications not recommended in pregnancy.^15^ This discrepancy illustrates the contrast in medication prescription patterns evident between women with type 2 diabetes and the broader female population of reproductive age, and further accentuates the need for proactive promotion of medication review within the type 2 diabetes population.

To date, few studies have explored the medication profile of women of reproductive age with type 2 diabetes, and to our knowledge, none have examined the medication exposure of this group with the level of granularity described here. Previous data captured in the National Pregnancy in Diabetes Audit (NPID) 2020–2022 report a prescription rate of 4% for statins, 4% for ACE inhibitors, and 7% for adverse diabetes medications in women with type 2 diabetes who became pregnant.^16^ The audit also reported that these agents are more common in women with type 2 diabetes compared with those with type 1 diabetes. Our data are aligned with these observations, as 19% and 17% of the women in our cohort not prescribed contraception by their GP were prescribed statins and/or RAS-acting agents, respectively. Additionally, when explored using a multivariable logistic regression model, age and number of medications prescribed were associated with increased odds of the use of medications not recommended for pregnancy. While it was unsurprising that the incidence of mediations not recommended in pregnancy was associated with polypharmacy, this association emphasizes the importance of prescribing in the context of a woman’s reproductive intention and use of contraception, and further underscores the urgency of medication review among women taking multiple medications. It also demonstrates that this population likely has a high comorbid burden, which contributes to the risk of adverse pregnancy outcomes.

As anticipated, the cohort was skewed towards older age, with a median age of approximately 38 years. While half of the cohort was aged 40 years or over, the pregnancy potential of this group should not be discounted. A cross-sectional study in the UK demonstrated that the use of unreliable or no contraception was highest among women aged 35–49 years, as compared with other age groups.^17^ Furthermore, between 2011 and 2021, conception rates in the UK among women aged 40 years and over grew by an estimated 20% (to 17.3 conceptions per 1000 women), reflecting a general nationwide trend of increasing maternal age.^18^ This trend, compounded by the rapidly growing population of younger women with type 2 diabetes, highlights the potential for additional unprepared high-risk pregnancies in this group without sufficient reproductive support and intervention.^19^,^21^ The inadequate recognition of the reproductive potential of women with type 2 diabetes due to their age and diabetes status has also been shown to impact pregnancy preparedness.^22^

Almost half (45%) of the cohort was of Black ethnic origin, with an additional 18% of Asian ethnic origin, reflecting the ethnic profile of the study area and the higher prevalence of type 2 diabetes in these populations.^3 23 24^ It was observed that white women were significantly more likely to be prescribed contraception by their GP than women of other ethnicities, a finding that suggests a higher potential for unplanned or unprepared pregnancy in Black and Asian women. This observation is particularly concerning given that women of Black and Asian ethnicity are less likely to receive pre-pregnancy care, undergo fewer antenatal checks, experience worse pregnancy outcomes, and have higher rates of maternal mortality when compared with white women, a reality in part attributable to persistent healthcare inequalities.^25^,^30^ A recent review of over 700 participants from ethnic minority groups, including Black and Asian communities, additionally found that contraception uptake is influenced by factors including harmful behaviors of healthcare professionals, as well as the significant influence of family, friends, and peers on contraception views and decision-making.^31^

This study also elucidates the extent to which women in this population meet specific criteria articulated in the NICE pre-pregnancy guidelines.^4^ Of the 89% of this cohort not using contraception prescribed by their GP, a high proportion did not meet current criteria, and none met all criteria. These findings echo the most recent data produced by NPID, which demonstrated that only 10% of women with pre-existing type 2 diabetes were prepared for pregnancy.^2 7^ However, one criterion in which our study data diverged from the audit data pertained to high dose folic acid use; 5% of the women in our cohort used high dose folic acid, as compared with 21% reported in the audit. This may be a further illustration that high dose folic acid is only prescribed when a woman’s pregnancy intention is known, again emphasizing the importance of eliciting pregnancy intention in primary care interactions. Prescriptions of high dose folic acid among women with type 2 diabetes in the UK lags behind that of women with type 1 diabetes who are managed by specialist diabetes teams where there is higher awareness of pre-pregnancy care; NPID data showed that 44% of women with type 1 diabetes are taking high dose folic acid prior to pregnancy.^16^

Glycemia and obesity were the most conspicuously unmet metabolic targets among this cohort. Only a third of women met the recommended pre-pregnancy HbA1c target (<48 mmol/mol or 6.5%), and a quarter of women had an HbA1c exceeding 69 mmol/mol (8.5%). While not all women in this cohort will become or intend to become pregnant, these findings are concordant with previous observations indicating that many with type 2 diabetes present at their first pregnancy booking above the current HbA1c target.^4^ This finding additionally emphasizes the importance of screening for reproductive intention and addressing possible risks of adverse pregnancy outcomes. Over 70% of the cohort were obese, a factor that is independently associated with increased risk of pregnancy complications and congenital defects.^23 24^ Here, our findings are broadly congruent with data on obesity in younger people with type 2 diabetes (<40 years of age), as reported in a recent UK national audit.^21^ High levels of obesity in women with reproductive potential emphasize the need to strengthen interdisciplinary primary care-based weight management resources consistent with NICE guidance available to this population.^3 4 32 33^ Evidence indicates that for obese individuals, a 5–10% reduction in BMI lowers the risk of preeclampsia, diabetes complications, and stillbirth.^34^ Discussing with women how weight management strategies relate to beneficial pregnancy outcomes could potentially improve motivation if undertaken in a constructive and collaborative manner.^35 36^

An unexpectedly high number of women in this cohort (14%) were prescribed at least one psychotropic medication, with antidepressants the most common of these prescriptions. Although our data concurs with other observational studies that report higher incidence of antidepressant use among women with type 2 diabetes as compared with women without diabetes (and men with type 2 diabetes),^37^,^40^ the use of antidepressants in this population warrants further study, particularly in relation to pregnancy outcomes. These data also highlight the need to support women’s mental health and well-being, as pregnancies with maternal diabetes are often associated with negative emotional thinking and identity disruption.^40 41^

Another unexpected observation was that those with a higher HbA1c (<48 mmol/L), were less likely to be on medications not recommended for pregnancy. This could be explained by multiple factors: the association between a greater number of diabetes therapies and improved glycemia; treatment avoidance behaviors leading to fewer medications among those with higher HbA1c; and the possibility that medications other than diabetes therapies, such as those used for the treatment of comorbidities, account for the association. A future prospective study could consider this association in more depth and investigate its interaction with diabetes therapies.

Polypharmacy was relatively common in the cohort, with 13% of women prescribed drugs from five or more different groups of medications. While many prescribed agents were related to diabetes, the medication profile and diversity of medication class suggested the potential presence of multimorbidity within the cohort.^42^ Increasing maternal age, rising obesity levels, and the expansion of diabetes therapies may contribute to the use of multiple medications during pregnancy, which has become increasingly prevalent.^43^ In a recent UK retrospective observational study, obesity, multimorbidity, and ethnicity were strongly associated with polypharmacy in pregnancy.^44^ An additional large cohort study conducted in the United States found that polypharmacy was related to obesity, although this relationship was only evident among selected medication classes such as diabetes medications and progesterone.^45^ Collectively, our data and the aforementioned studies underscore the significance of polypharmacy and multimorbidity in women of reproductive age with diabetes. These factors are often overlooked in younger cohorts and demand additional attention. In the UK, the NHS’s new programe, *T2Day: Type 2 Diabetes in the Young*, may help address some of these concerns, as it aims to enhance pregnancy awareness and promote medication review in people with type 2 diabetes under 40 years of age. The effectiveness of this programe, however, has yet to be established.^46^ Overall, these findings indicate the importance of developing more responsive reproductive care for women with type 2 diabetes. This will require quality improvement initiatives and system changes to ensure that the needs of this population are more adequately addressed.

The limitations to our analysis must be considered in the interpretation of our findings. First, while previous research indicates that GPs remain the primary means by which women obtain contraception in the UK, women can access contraception from a variety of other sources, such as reproductive health clinics and pharmacies.^47 48^ Similarly, electronic medical record data is limited in its inability to capture use of barrier contraception, practice of abstinence and/or the rhythm method, and infertility unrelated to documented gynecological surgery. These factors likely contribute to an underestimation of contraception use within this cohort. Additionally, as the data were extracted directly from electronic patient records, we could not control data completeness. We encountered missing data for several clinical variables (urine albumin to creatinine ratio and parity) and, to avoid compromising the quality of subsequent analyses, we removed these variables. We were similarly dependent on the correct coding of type 2 diabetes by medical practitioners in primary care settings. Due to the lack of data on medication adherence, we were unable to confirm whether prescriptions translated into actual medication use. Previous research indicates that suboptimal adherence remains a persistent issue among individuals living with type 2 diabetes in the UK^49^; however, further investigation is particularly needed among women of reproductive age. Nevertheless, this limitation does not compromise our assessment of current pregnancy preparedness levels in this population. The inclusion of additional sociodemographic variables such as socioeconomic deprivation, which has been shown to have a striking interaction with pregnancy in women with type 2 diabetes, could also improve future studies.^7^ More studies in a wider range of contexts need to be undertaken to establish the national and international generalizability of our observations and should include prospective cohort studies to fully estimate the association between medications and pregnancy outcomes. Finally, this analysis lacked a UK-specific medication categorization system to establish potential medication hazards. Our research team developed an appropriate method of classification based on BNF guidance, but our study highlights the potential value of a comprehensive UK-specific medication categorization system for pregnancy safety. While the UK Teratology Information Service is a useful resource, its scope did not sufficiently account for the diversity of medications prescribed to this cohort.

## Conclusions

The results of this study suggest that women of reproductive age with type 2 diabetes have multiple exposures associated with negative pregnancy outcomes. Prior to pregnancy, a large proportion of women do not take high dose folic acid, exhibit suboptimal glycemia, and continue medications that are not recommended for or are hazardous in pregnancy. The implications of these concerns are potentially exacerbated by the prevalence of multimorbidity, polypharmacy, and obesity, in addition to the low utilization of adequate contraception. Collectively, our findings emphasize the need for the consistent assessment of the reproductive intentions of women with type 2 diabetes in primary care settings, followed by the provision of either acceptable contraception or appropriate pre-pregnancy advice and intervention incorporating a comprehensive medication review.

## supplementary material

10.1136/bmjdrc-2024-004312 online supplemental file 1

10.1136/bmjdrc-2024-004312 online supplemental file 2

## Data Availability

Data are available on reasonable request.
