# Supplementary material for: Medication prescribing and pregnancy-related risk factors for women with type 2 diabetes of reproductive age within primary care: a cross-sectional investigation for the PREPARED study
Source: BMJ Open Diabetes Res Care. 2025 Mar 5;13(2):e004312. doi: 10.1136/bmjdrc-2024-004312 (PMC11883600; doi:10.1136/bmjdrc-2024-004312)
Supplement: online supplemental file 2 [file bmjdrc-13-2-s002.pdf]

Table S2. Diabetes medication by HbA1c category among women not prescribed contraception by their general practitioner

Diabetes medications	<48 mmol/mol (n=180)	48-57 mmol/mol (n=199)	58-69 mmol/mol (n=76)	70-85 mmol/mol (n=70)	≥86 mmol/mol (n=111)
DPP-4 inhibitors	9 (5.0)	10 (5.0)	10 (13.2)	7 (10.0)	15 (13.5)
GLP-1 inhibitors	6 (3.3)	4 (2.0)	4 (5.3)	5 (7.1)	9 (8.1)
Insulin	12 (6.7)	15 (7.5)	14 (18.4)	14 (20.0)	38 (34.2)
Metformin	78 (43.3)	109 (54.8)	55 (72.4)	51 (72.3)	72 (64.9)
Secretagogues	6 (3.3)	9 (4.5)	10 (13.2)	14 (20.0)	34 (30.6)
SGLT2 inhibitors	7 (3.9)	11 (5.5)	10 (13.2)	8 (11.4)	11 (9.9)

Values are presented as n (%), with % indicating the proportion of women within the HbA1c category that have been prescribed the stated diabetes medication. Percentages in each column do not add up to 100, as women may have been prescribed multiple medications. DPP-4 = dipeptidyl peptidase-4; GLP-1 = glucagon-like peptide-1; SGLT2 = sodium-glucose co-transporter-2.
